# Clinical Value of Tissue Transglutaminase Antibodies in Celiac Patients over a Long Term Follow-Up

**DOI:** 10.3390/nu13093057

**Published:** 2021-08-31

**Authors:** Elisa Farina, Leda Roncoroni, Vincenza Lombardo, Alice Scricciolo, Maurizio Vecchi, Luisa Doneda, Luca Elli

**Affiliations:** 1 Department of Pathophysiology and Transplantation, University of Milan, 20122 Milan, Italy; elisa.farina@unimi.it (E.F.); maurizio.vecchi@unimi.it (M.V.); 2 Foundation IRCCS Ca’ Granda Ospedale Maggiore Policlinico—Center for Prevention and Diagnosis of Celiac Disease and Division of Gastroenterology and Endoscopy, Via F. Sforza 35, 20122 Milan, Italy; leda.Roncoroni@unimi.it (L.R.); vincenza.lombardo@policlinico.mi.it (V.L.); scricciolo.alice@gmail.com (A.S.); 3 Department of Biomedical, Surgical and Dental Sciences, University of Milan, 20133 Milan, Italy; luisa.doneda@unimi.it

**Keywords:** celiac disease, anti-tissue transglutaminase, urinary gluten detect

## Abstract

Introduction & Aim: Anti-tissue transglutaminase antibody (tTGA) titer is used during the follow-up of celiac patients to evaluate gluten-free diet (GFD) responsiveness. However, no clear data are available on this issue. The aim of this study was to evaluate tTGA significance during celiac disease (CD) monitoring. Methods: From January 2017 to January 2020, consecutive CD patients on a GFD with persistent positive tTGA were enrolled. Antibody titres were evaluated on a yearly basis from CD diagnosis to the last follow-up. Urinary gluten detection tests, duodenal histology and capsule enteroscopy (CE) were performed. A tTGA-positive cohort was compared with a control group composed of 212 treated CD patients with negative tTGA. Results: 65 patients (12% males, median age at enrollment and CD diagnosis, 37 (14–86) and 31 (1–76), respectively, median follow up 4 (1–26) years) presented with positive tTGA during follow-up. Overall, the tTGA titres were 3 (1–79) fold increased (ULN). Three different tTGA trends were recognized: (I) 36 (55%) patients with a progressive titres decrease; (II) 16 (25%) patients with a fluctuating behavior; (III) 13 (20%) patients with a steady state or increased titres. tTGA+ patients did not present with different clinical and demographic parameters. Duodenal atrophy was present in 10% vs. 36% of the tTGA positive vs. negative group (*p* < 0.005), respectively. Gluten detection results were positive in 3 (8%) cases, all in the III group. In tTGA+ patients, CE did not identify any CD-related complications. Conclusions: tTGA positivity during CD follow up did not present a relevant clinical significance without association with autoimmune comorbidities and mucosal damage.

## 1. Introduction

Celiac disease (CD) is a chronic, multi-organ autoimmune disease that affects the small bowel in genetically predisposed subjects, precipitated by the ingestion of gluten [1]. Based on serological screening, the worldwide prevalence of CD is 1.4%, with prevalence dropping to 0.7% in the event of histological analysis [2]. Available data suggest that CD incidence is truly increasing and that the disease is currently much more common in some areas than previously reported [3]. The diagnosis of CD is variably defined by four components: symptoms; the presence of HLA-DQ2/DQ8; autoimmune antibodies in serum; and duodenal histology [4,5,6,7]. Anti-tissue transglutaminase antibody (tTGA) is the preferred single test for detection or screening of CD at any age. The sensitivity of tTGA for untreated CD is about 95%; the specificity is also 95% or greater. The higher the titer of the test, the greater the likelihood of a true positive result [5]. tTGA is the most sensitive test for CD, whereas IgA-EMA is the most specific one. IgA-EMA may be used as a confirmatory test, particularly when tTGA has a low titer (<2 times the upper normal limit (ULN)) [1]. The antibodies directed against gliadin or its deamidated products, as well as the self-antigen tTGA, are dependent on the ingestion of gluten. The reduction or total elimination of dietary gluten leads to a decrease in the levels of antibodies directed against gliadin or tTGA. A weakly positive antibody titer may become negative within weeks of strict adherence to a gluten-free diet (GFD). After 6–12 months of adhering to a GFD, 80% of subjects will test negative by serology. By five years, more than 90% of those adhering to the GFD have negative serology [8]. The usefulness of tTGA for the diagnosis of CD is well-known, but their role in follow-up remains unclear. However, tTGA are widely used in disease monitoring, but their true significance in clinical practice is not known. In spite of this uncertain significance, routine tTGA testing is recommended in CD monitoring to evaluate GFD adherence and CD remission activity [4,9,10]. Thus, in clinical practice, tTGA are used as an alarm biomarker suggesting a complicated disease, although this is not evidence-based. As a consequence, how to follow and monitor patients with persistently positive tTGA over time and the clinical significance of this finding represents a challenge in daily practice. Frequently, tTGA positivity is followed by a number of invasive investigations, such as endoscopy, to obtain histology samples, and videocapsule endoscopy (VCE), to evaluate the entire small bowel mucosa. However, no clear information is available in the guidelines about a long-term strategy. The aim of our study was to analyze the clinical significance of tTGA antibodies in a cohort of treated CD patients with persistently positive serology.

## 2. Materials and Methods

### 2.1. Study Design and Patients

From January 2017 to January 2020, consecutive CD patients attending the Center for the Prevention and Diagnosis of CD—Gastroenterology and Endoscopy Unit, Fondazione IRCCS Ca’ Granda Ospedale Maggiore Policlinico (Milan, Italy) were evaluated and consecutively enrolled in the study. Inclusion criteria were adult CD patients with persistent positive tTGA despite a correct adherence to a GFD. Diagnosis of CD was based upon national and international guidelines [1,5,11]. Clinical and demographic data from the time of CD diagnosis to study enrollment were evaluated, such as tTGA titres (evaluated yearly), adherence to the diet (evaluated by means of nutritionist interview, celiac dietary adherence test (CDAT) and urinary gluten immunologic peptides (GIP)), duodenal histology and capsule, and, eventually, double balloon enteroscopy when available [12]. The CD onset was classified as: classical (diarrhea, weight loss, longitudinal growth retardation), non-classical (dyspepsia, anemia, hypertransaminasemia, osteopenia, etc.), associated with the presence of dermatitis herpetiformis or during a screening program on first-degree relatives [13]. A group of celiac patients fully adherent to the GFD with negative antibodies were used as controls.

The study was approved by the local ethics committee and was carried out in accordance with the Declaration of Helsinki.

### 2.2. Antibodies, Endoscopy and Histological Analysis

tTGA were evaluated by means of commercially available ELISA kits, following the manufacturer’s instructions. tTGA values have been expressed as folds upper the normal limits (ULN). Patients undergoing SB examination were evaluated by means of capsule endoscopy (CE) (PillCam SB3, Medtronic, Dublin, Ireland). CE investigations were performed as previously described [14,15,16,17]. The day before CE, patients were administered with 2 L polyethylene glycol and overnight fasting for SB cleaning. SB preparation was evaluated in accordance with Brotz et al. [18]. The videos were reviewed by one expert operator (L.E., reading more than 100 CE per year). Mean extensions of the lesions were detected and expressed as a percentage of the total transit time of the capsule in the SB. In particular, mucosal atrophy was defined by the presence of scalloped folds, fissures or a mosaic pattern in the mucosa, granular mucosa and a reduction or absence of duodenal Kerckring’s folds, as previously described [19]. Furthermore, during follow-up, patients underwent upper endoscopy with duodenal sampling (four oriented biopsies in the distal duodenum and two in the bulb). Duodenal histology was classified according to the Marsh–Oberhuber grading [20] and used to evaluate the sensitivity and specificity of CE for SB atrophy.

### 2.3. Urine Samplings

CD patients with positive tTGA were given a sterile urine container and asked for a sample. This was analyzed in the laboratory the same day to prevent degradation of any peptides present. The GlutenDetect kit (Biomedal S.L., Sevilla, Spain) was used to search for the presence of GIP. In addition to the 33-mer peptide, this methodology also recognizes similar peptides that react with the G12 monoclonal antibody [21].

### 2.4. Statistical Analysis

The data were statistically analyzed using STATA software (release 10.0, Stata Corporation, College Station, TX, USA). Patients’ characteristics are described by means of relative frequencies and percentage for qualitative variables, and by median and range for quantitative variables. Wilcoxon rank-sum test was used to compare values in two groups of patients, according to tTGA positive status. A *p*-value < 0.05 was considered statistically significant. The study was approved by the local ethical committee (n 19-2019bis).

## 3. Results

### 3.1. Clinical and Demographic Findings

The demographic and clinical features of 65 CD patients with persistently positive tTGA values and 212 CD patients with negative tTGA on a GFD are reported in Table 1. 

**Figure 1:**
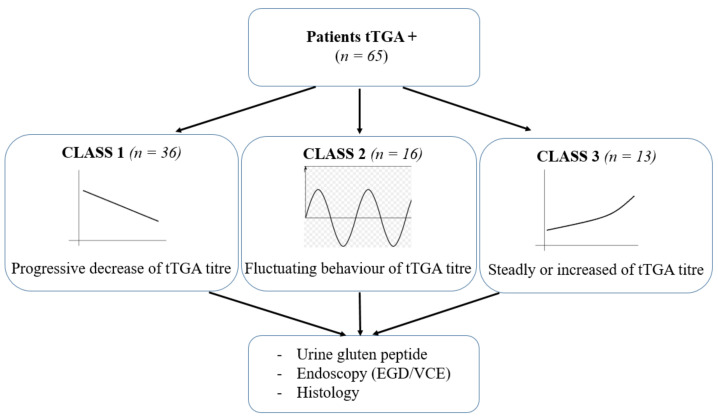
Workflow and distribution of patients following their tTGA trend.

Overall, the increase of tTGA was 3 (1–79)-fold the ULN; the median follow-up was 4 (1–26) years. The two groups (positive and negative tTGA) did not differ significantly in age, gender, associated autoimmune comorbidities, and age at CD onset. Regarding the phenotype at CD diagnosis, patients in the control group presented more frequently with a classic clinical picture, while patients maintaining a positive value of tTGA were more frequently characterized by a mono-paucisymptomatic picture or were diagnosed during screening programs for CD-associated diseases. Urinary GIP resulted positive in three (8%) of the tTGA-positive cases. CDAT was not related either the GIP result or tTGA titer. Looking at the trend of tTGA during the follow-up, three different classes of patients were recognized (Figure 1). 

**Figure 2:**
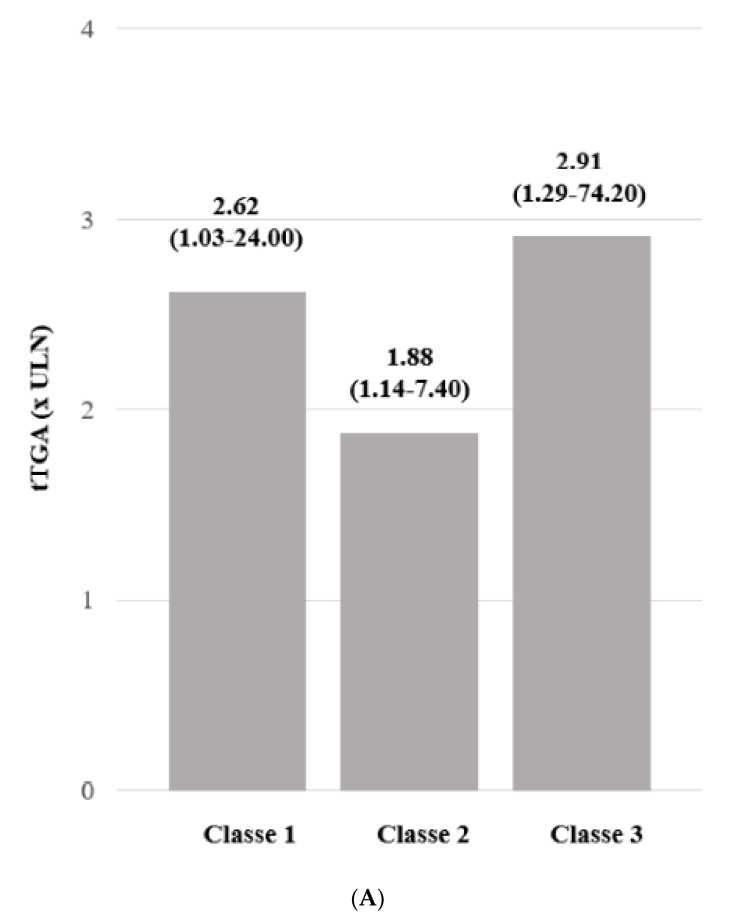

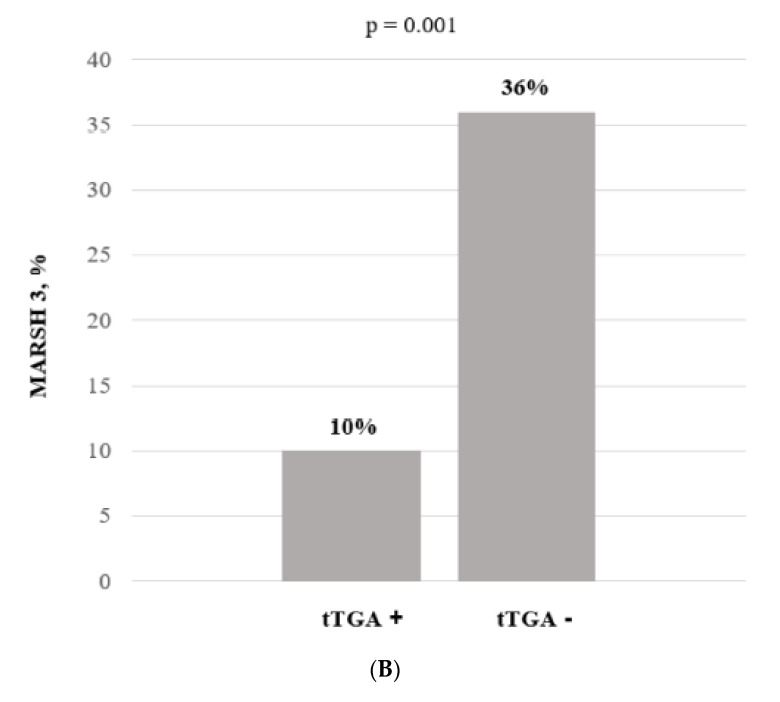
(**A**): tTGA levels (folds increased above ULN) during follow-up in the three classes; (**B**): Percentage of patients with Marsh grade 3 at follow-up endoscopy.

The first group (Class 1) of patients, composed of 36 (55%) patients, was characterized by a progressive decrease in tTGA titres during 3 (1–13) years of follow-up. Eight (22%) patients became negative after 5.6 ± 4.0 years post-CD diagnosis. The second group (Class 2, 16 (25%) CD patients) showed a fluctuating behavior during the 7 (1–26) years of follow-up. Eight (50%) patients in this class had antibody titres that turned negative at least once during the time of follow-up. In the third group (Class 3), 13 (20%) patients presented with a steady or increased titer of tTGA after CD diagnosis. The follow-up was 9 (2–19) years. All three patients with positive urinary GIP belonged to this class. The tTGA levels were not statistically different between Classes 1, 2 and 3 (Figure 2A). The clinical and demographic characteristics of the three classes of tTGA positive patients are reported in Table 2.

### 3.2. Endoscopic and Histologic Findings

The second duodenal histology profile of the tTGA positive and negative groups is shown in Figure 2B; this evidenced a significant difference between tTGA positive and negative patients. Considering the tTGA trends (Classes 1, 2 and 3), the duodenal histology patterns did not present statistically significant differences (Table 2).

**Figure 3:**
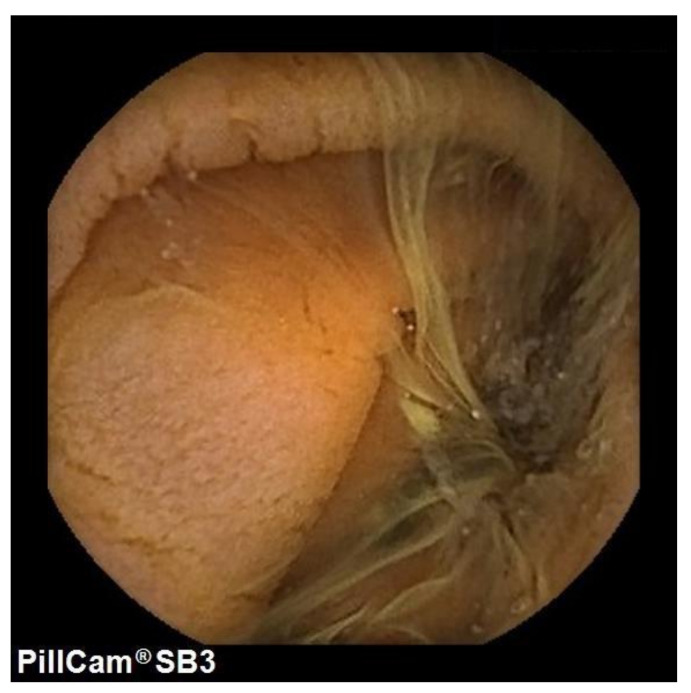
Mucosa atrophy of the small bowel.

In the tTGA positive group, 24 out 65 patients underwent VCE, and in 42% mucosal atrophy was detected, mainly in the proximal part of the small bowel (Figure 3). The percentage of patients with small bowel presenting with atrophy at VCE was 14% (5–40%). Notably, in only 3 (12.5%) cases, atrophy was histologically confirmed.

## 4. Discussion

In our study, we demonstrated that different trends of tTGA can be evidenced during CD follow-up: patients with a decreasing titer, patients with a fluctuating titer, and patients with an increasing titer over time. Our findings showed that only in the latter scenario does the positivity of tTGA present with clinical significance by being related to unconscious gluten intake in the diet, as demonstrated by urinary GIP. Furthermore, tTGA values did not correlate with questionnaires, histological disease activity and CD phenotype. 

CD is a chronic inflammatory condition, and persistence of the inflammatory state may lead to complications, including nutritional deficiencies, osteoporosis and increased risk of certain types of cancer [23]. There are no guidelines for CD monitoring, and parameters to evaluate routinely during follow-up are not validated. However, tTGA titres are currently used to evaluate the adherence and responsiveness to a GFD and usually checked yearly in daily clinical practice. It is well-known that compliance with a GFD will lead to disease control in a great majority of patients with CD, and consequently, decrease the risk of complications and mortality [24]. 

Our study analysed a group of CD subjects with positive tTGA who reported following a correct GFD; these patients were compared to a control group of CD subjects on a correct GFD with negative tTGA during follow-up. We did not find any difference regarding age at diagnosis and at baseline in these two groups; notably, subjects with persistently positive tTGA had a mono-paucisymptomatic clinical picture at CD diagnosis or were diagnosed during screening programs for CD-associated diseases [22,25]. We focalized our attention on tTGA trend more than on single results; this was made possible by the long-term follow-up of our patients (4, 1–26). Looking at the tTGA titer during follow-up, it is possible to recognized three different patterns. In the first class (55%) of patients, we identified subjects with a slow progressive decrease of the titer, the so-called slow responder [26]. In the second class (25%) of patients, we appreciated a fluctuating behaviour; half of the patients in this class had the antibody titresthat turned negative at least once during the time of follow-up. The last class (20%) of patients was characterized by a steady or increased titer during follow-up. In our study, there are no significant baseline differences between the three classes. Only subjects included in this last class who presented with a steady or increased tTGA titer during follow-up despite reporting a correct GFD presented with positive urinary GIP, thus demonstrating an unconscious ingestion of gluten in the diet.

CD follow-up is controversial and serological response is often used as a surrogate for histological recovery and GFD adherence [26]. In the literature, there are many studies that show that a complete recovery of the intestinal mucosa occurs rarely in celiac patients on a GFD, and, furthermore, tTGA are not useful predictors of mucosal healing, if mucosal healing represents the reference standard to evaluate the activity or remission of CD [27,28,29,30,31]. Surprisingly, patients with tTGA+ titres presented a lower rate of mucosal atrophy compared to the control group. This finding could be due to the low sensitivity of tTGA for small bowel atrophy, as previously reported by other studies; the percentage of CD patients presenting with atrophy during follow-up in the control group in line with that previously reported in literature [32,33,34,35]. Furthermore, a subgroup of patients was analyzed by means of CE. Enteroscopy suggested the presence of atrophy in roughly half of the cases with tTGA positivity. However, most of these cases were not confirmed by histology. This could be due to the lack of sensitivity of CE in detecting lesions of the very proximal part of the small bowel (bulb and duodenum), even though it remains a valuable technique to exclude the presence of dangerous CD complications along the small bowel (ie intestinal lymphoma, ulcerative jejunoileitis and carcinoma) [19,36,37,38].

To the best of our knowledge, this is the first study to analyze a cohort of patients with positive tTGA and monitor trends in their tTGA titer during a long follow-up. Furthermore, this is the first study to use CE to monitor CD; we did not find any CD complication in tTGA+ patients over a long-term follow-up period [39]. In spite of the relevant findings of the study, some limitations can be drawn; namely, the study enrolled patients from a tertiary referral setting and, thus, the cohort could be affected by selection bias. If the single center setting confers homogeneity to the follow-up of the patients (evaluated on an yearly basis), on the other side, clinical approaches could be different in other centers and other realities.

## 5. Conclusions

Our study casts doubts and shadows on the real need to monitor patients’ tTGA values to define their CD state. A slowly decreasing or fluctuating trend does not frighten clinicians and patients, though particular attention should be reserved to patients with an increasing titre, even though the real prognostic value of unconscious gluten introduction is still under debate [40,41].

## Figures and Tables

**Table 1:** Baseline features of CD patients with positive and negative tTGA.

Baseline Variables	CD Patients with IgA-TG2 Positive (*n* = 65)	CD Patients with Negative IgA-TG2 (*n* = 212)	*p*-Value Time Independent
Age at diagnosis, years *	31 (1–76)	29 (1–67)	0.659
Age at enrollment, years *	37 (14–86)	39 (16–78)	0.015
Male, *n* (%)	8 (12%)	38 (18%)	0.256
Autoimmune disease, *n* (%)	13 (20%)	52 (25%)	0.409
**Onset**, ***n* (%)**			
Infancy °	5 (8%)	12 (6%)	0.567
Pediatric °°	8 (12%)	32 (15%)	0.546
Adult ^	52 (80%)	161 (76%)	0.504
Elderly ^§^	0	7 (3%)	0.159
**Phenotype**, ***n* (%)**			
Classic	15 (23%)	111 (52%)	<0.001
Mono-paucisymptomatic	35 (54%)	84 (40%)	0.047
Dermatitis herpetiformis	4 (6%)	4 (2%)	0.095
Familiar screening	5 (8%)	8 (4%)	0.194
Screening for other diseases	6 (9%)	5 (2%)	0.009

* median (range); ° infancy ≤ 2 years; °° pediatric > 2 and ≤14 years; ^ adult > 14 and ≤60 years; ^§^ elderly > 60 years [22].

**Table 2:** Baseline features of CD patients with positive tTGA divided in the three classes.

Baseline Variables	Class 1 (*n* = 36)	Class 2 (*n* = 16)	Class 3 (*n* = 13)	*p*-Value
Age at diagnosis, years *	34 (14–76)	31 (1–54)	30 (5–56)	0.425
Age at enrolling, years *	37 (17–86)	37 (20–63)	37 (14–69)	0.975
Male, *n* (%)	5 (14%)	1 (7%)	2 (15%)	0.690
Autoimmune disease, *n* (%)	6 (17%)	4 (33%)	3 (23%)	0.749
**Onset**, ***n* (%)**				0.104
Pediatric	0	3 (19%)	2 (15%)	0.033
Teenage	6 (17%)	1 (6%)	1 (8%)	0.488
Adult	30 (83%)	12 (75%)	10 (77%)	0.749
Elderly	0	0	0	-
**Onset fenotype, *n* (%)**				0.640
Classic	6 (17%)	5 (3%)	4 (31%)	0.393
Mono-paucisymptomatic	23 (65%)	6 (38%)	6 (46%)	0.175
Dermatitis herpetiformis	3 (8%)	1 (6%)	0	0.563
Familiar screening	2 (5%)	2 (12%)	1 (8%)	0.686
Screening for other diseases	2 (5%)	2 (12%)	2 (15%)	0.504
TTGA titer, (x UNL)	3 (1–24)	2 (1–7)	3 (1–74)	0.314
Follow-up endoscopy available	15 (42%)	12 (75%)	12 (92%)	0.002
Marsh 3 at follow-up endoscopy, *n* (%)	2 (13%)	0	2 (17%)	0.357

* median (range).

## Data Availability

No new data were created or analyzed in this study. Data sharing is not applicable to this article.
